# The genome sequence of the Oak Hook-tip, *Watsonalla binaria *(Hufnagel, 1767)

**DOI:** 10.12688/wellcomeopenres.19634.1

**Published:** 2023-07-26

**Authors:** Douglas Boyes, Physilia Chua, University of Oxford and Wytham Woods Genome Acquisition Lab, Darwin Tree of Life Barcoding collective, Wellcome Sanger Institute Tree of Life programme, Wellcome Sanger Institute Scientific Operations: DNA Pipelines collective, Tree of Life Core Informatics collective, Darwin Tree of Life Consortium

**Affiliations:** 1 UK Centre for Ecology & Hydrology, Wallingford, England, UK; 2 Wellcome Sanger Institute, Hinxton, England, UK

**Keywords:** Watsonalla binaria, Oak Hook-tip, genome sequence, chromosomal, Lepidoptera

## Abstract

We present a genome assembly from an individual female *Watsonalla binaria* (the Oak Hook-tip; Arthropoda; Insecta; Lepidoptera; Drepanidae). The genome sequence is 333.0 megabases in span. Most of the assembly is scaffolded into 33 chromosomal pseudomolecules, including the W and Z sex chromosomes. The mitochondrial genome has also been assembled and is 15.24 kilobases in length. Gene annotation of this assembly on Ensembl identified 16,002 protein coding genes.

## Species taxonomy

Eukaryota; Metazoa; Eumetazoa; Bilateria; Protostomia; Ecdysozoa; Panarthropoda; Arthropoda; Mandibulata; Pancrustacea; Hexapoda; Insecta; Dicondylia; Pterygota; Neoptera; Endopterygota; Amphiesmenoptera; Lepidoptera; Glossata; Neolepidoptera; Heteroneura; Ditrysia; Obtectomera; Drepanoidea; Drepanidae; Drepaninae; Watsonalla (Hufnagel, 1767) (NCBI:txid721165).

## Background

The Oak Hook-tip, *Watsonalla binaria*, is a species of moth from the family Drepanidae, or ‘Hook-tip’ moths. This family gets its name from the shape of its tip at the forewings ( Kimber, 2023). One of the smallest members of Drepanidae, *W. binaria* has a wingspan of 18 to 30 mm. Some distinctive features used for identification are: two pale cross-lines on its orange-brown forewings, two prominent twin dark spots on its forewings, and two central hindwing spots ( Lewis, 2020).

The Oak Hook-tip is attracted to light and is mainly nocturnal. It can be found across most of Europe and is quite common in the UK, except for Scotland. Its main habitats are oak woodland and parkland, where oak is the primary food source for the larva, although it has been documented to feed on alder, beech, and birch ( NatureSpot, 2022). The species overwinters as a pupa. Two broods are usually produced, one flying in May and June, and the second flying in August. The second brood of moths are smaller and lighter in colour.

The Oak Hook-tip is listed as “vulnerable” with continued steep population decline ( Fox *et al.*, 2019).

The genome of the Oak Hook-tip was sequenced as part of the Darwin Tree of Life Project, a collaborative effort to sequence all named eukaryotic species in the Atlantic Archipelago of Britain and Ireland.

## Genome sequence report

**Figure 1.**
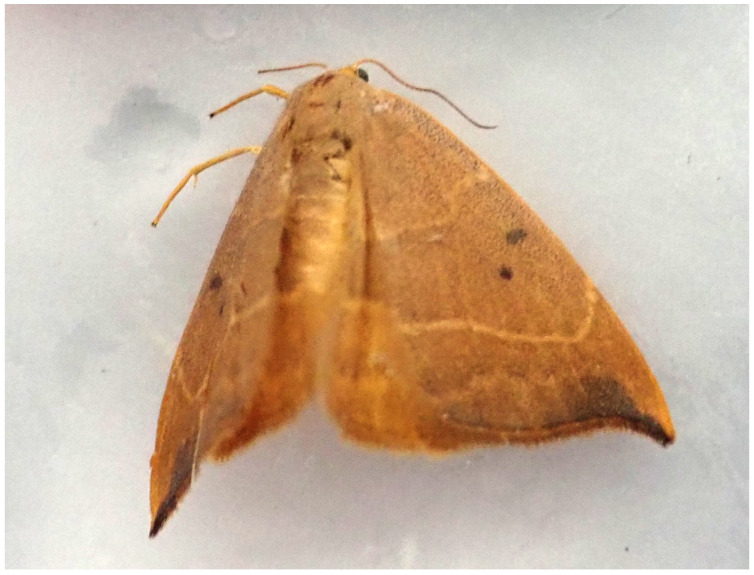
Photograph of the *Watsonalla binaria* (ilWatBina1) specimen used for genome sequencing.

The genome was sequenced from one female *Watsonalla binaria* ( Figure 1) collected from Wytham Woods, Oxfordshire, UK (51.77, –1.34). A total of 70-fold coverage in Pacific Biosciences single-molecule HiFi long reads and 102-fold coverage in 10X Genomics read clouds were generated. Primary assembly contigs were scaffolded with chromosome conformation Hi-C data. Manual assembly curation corrected 7 missing joins or mis-joins, reducing the scaffold number by 12.2%.

**Figure 2.**
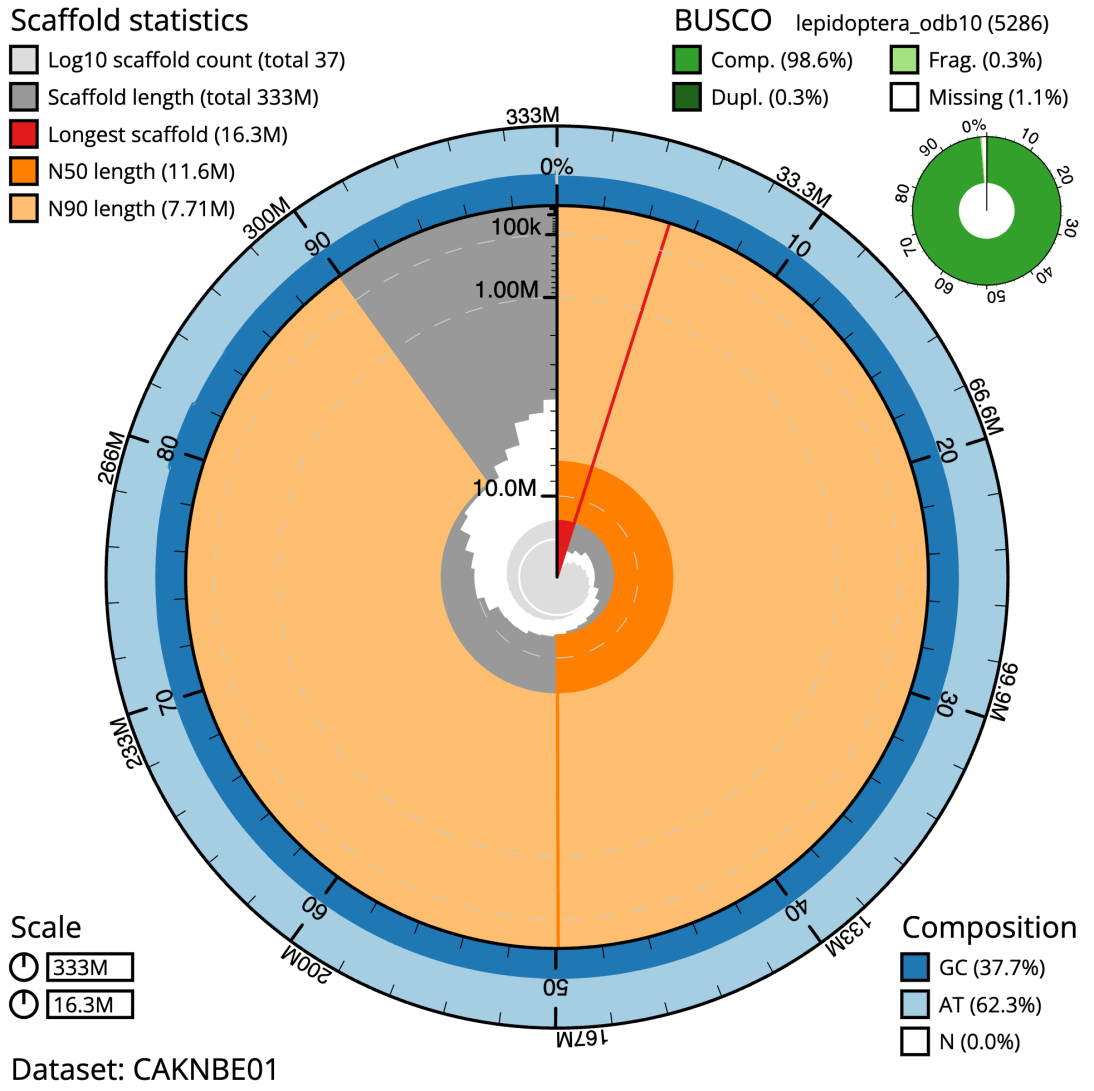
Genome assembly of *Watsonalla binaria*, ilWatBina1.1: metrics. The BlobToolKit Snailplot shows N50 metrics and BUSCO gene completeness. The main plot is divided into 1,000 size-ordered bins around the circumference with each bin representing 0.1% of the 333,062,894 bp assembly. The distribution of scaffold lengths is shown in dark grey with the plot radius scaled to the longest scaffold present in the assembly (16,345,438 bp, shown in red). Orange and pale-orange arcs show the N50 and N90 scaffold lengths (11,581,856 and 7,711,609 bp), respectively. The pale grey spiral shows the cumulative scaffold count on a log scale with white scale lines showing successive orders of magnitude. The blue and pale-blue area around the outside of the plot shows the distribution of GC, AT and N percentages in the same bins as the inner plot. A summary of complete, fragmented, duplicated and missing BUSCO genes in the lepidoptera_odb10 set is shown in the top right. An interactive version of this figure is available at https://blobtoolkit.genomehubs.org/view/ilWatBina1.1/dataset/CAKNBE01/snail.

The final assembly has a total length of 333.0 Mb in 36 sequence scaffolds with a scaffold N50 of 11.6 Mb ( Table 1). Most (99.97%) of the assembly sequence was assigned to 33 chromosomal-level scaffolds, representing 31 autosomes and the W and Z sex chromosomes. Chromosome-scale scaffolds confirmed by the Hi-C data are named in order of size ( Figure 2– Figure 5; Table 2). While not fully phased, the assembly deposited is of one haplotype. Contigs corresponding to the second haplotype have also been deposited. The mitochondrial genome was also assembled and can be found as a contig within the multifasta file of the genome submission.

**Table 1.** Genome data for *Watsonalla binaria*, ilWatBina1.1.

Project accession data		
Assembly identifier	ilWatBina1.1	
Species	*Watsonalla binaria*	
Specimen	ilWatBina1	
NCBI taxonomy ID	721165	
BioProject	PRJEB48674	
BioSample ID	SAMEA7746618	
Isolate information	ilWatBina1, abdomen (DNA sequencing), head and thorax (Hi-C scaffolding)	
Assembly metrics *		*Benchmark*
Consensus quality (QV)	60.5	*≥ 50*
*k*-mer completeness	100%	*≥ 95%*
BUSCO **	C:98.6%[S:98.3%,D:0.3%], F:0.3%,M:1.1%,n:5286	*C ≥ 95%*
Percentage of assembly mapped to chromosomes	99.97%	*≥ 95%*
Sex chromosomes	Z and W chromosomes	*localised homologous pairs*
Organelles	Mitochondrial genome assembled	*complete single alleles*
Raw data accessions		
PacificBiosciences SEQUEL II	ERR7419407	
10X Genomics Illumina	ERR7337604–ERR7337607	
Hi-C Illumina	ERR7337608	
Genome assembly		
Assembly accession	GCA_929442735.1	
*Accession of alternate haplotype*	GCA_929442715.1	
Span (Mb)	333.0	
Number of contigs	44	
Contig N50 length (Mb)	11.6	
Number of scaffolds	36	
Scaffold N50 length (Mb)	11.6	
Longest scaffold (Mb)	16.4	
Genome annotation		
Number of protein-coding genes	16,002	
Number of gene transcripts	16,183	

* Assembly metric benchmarks are adapted from column VGP-2020 of “Table 1: Proposed standards and metrics for defining genome assembly quality” from ( Rhie *et al.*, 2021). **BUSCO scores based on the lepidoptera_odb10 BUSCO set using v5.3.2. C = complete [S = single copy, D = duplicated], F = fragmented, M = missing, n = number of orthologues in comparison. A full set of BUSCO scores is available at https://blobtoolkit.genomehubs.org/view/ilWatBina1.1/dataset/CAKNBE01/busco.

**Figure 3.**
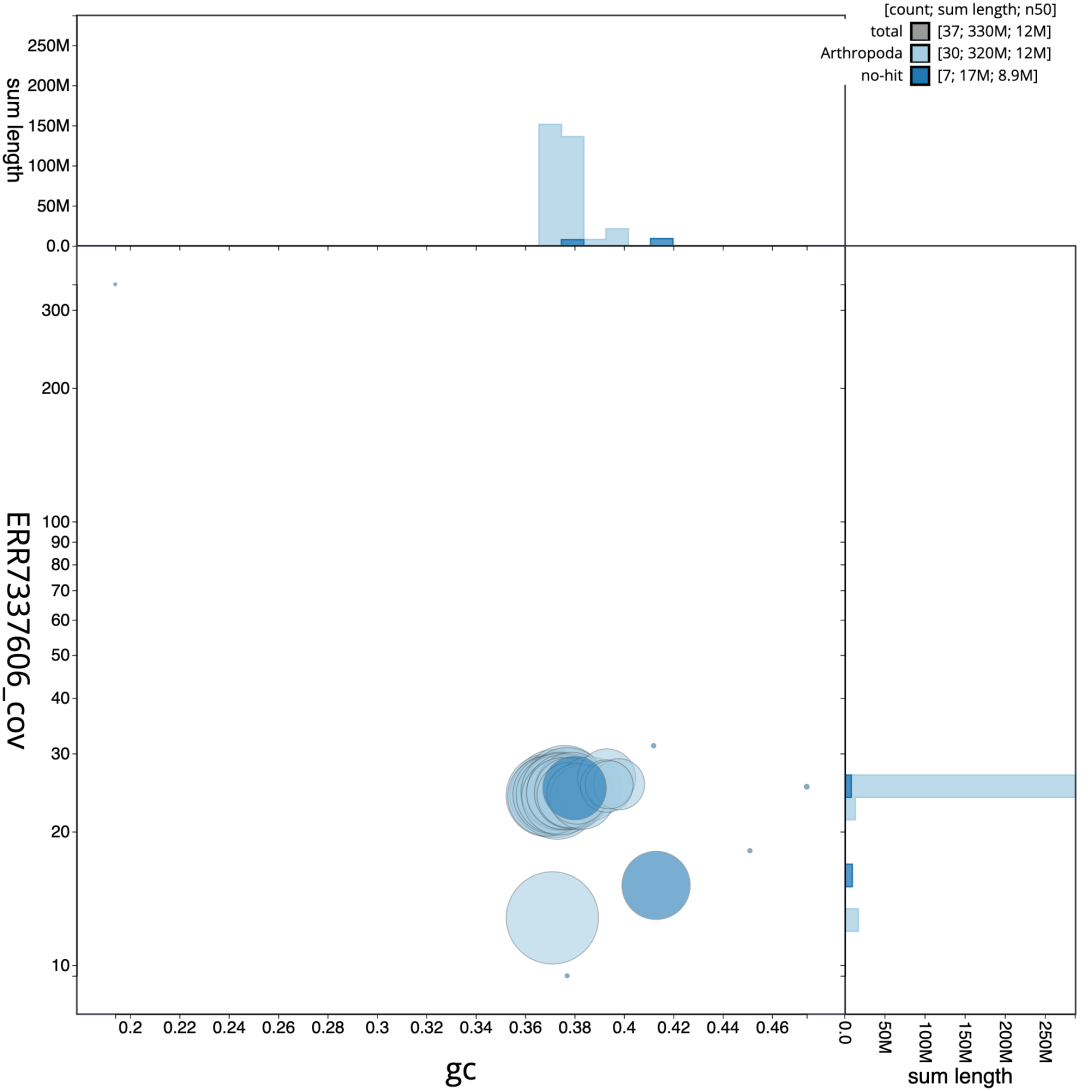
Genome assembly of *Watsonalla binaria*, ilWatBina1.1: BlobToolKit GC-coverage plot. Scaffolds are coloured by phylum. Circles are sized in proportion to scaffold length. Histograms show the distribution of scaffold length sum along each axis. An interactive version of this figure is available at https://blobtoolkit.genomehubs.org/view/ilWatBina1.1/dataset/CAKNBE01/blob.

**Figure 4.**
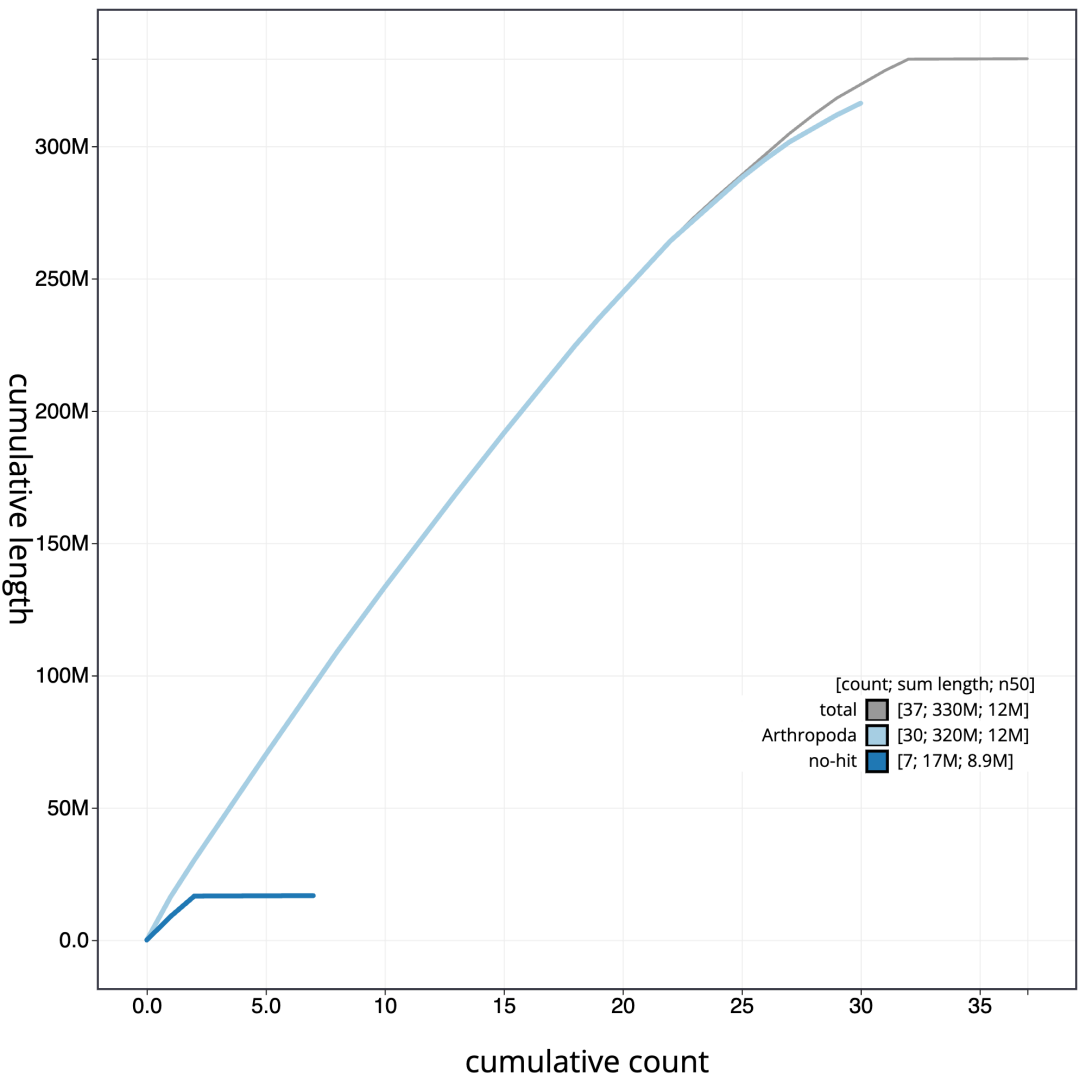
Genome assembly of *Watsonalla binaria*, ilWatBina1.1:BlobToolKit cumulative sequence plot. The grey line shows cumulative length for all scaffolds. Coloured lines show cumulative lengths of scaffolds assigned to each phylum using the buscogenes taxrule. An interactive version of this figure is available at https://blobtoolkit.genomehubs.org/view/ilWatBina1.1/dataset/CAKNBE01/cumulative.

**Figure 5.**
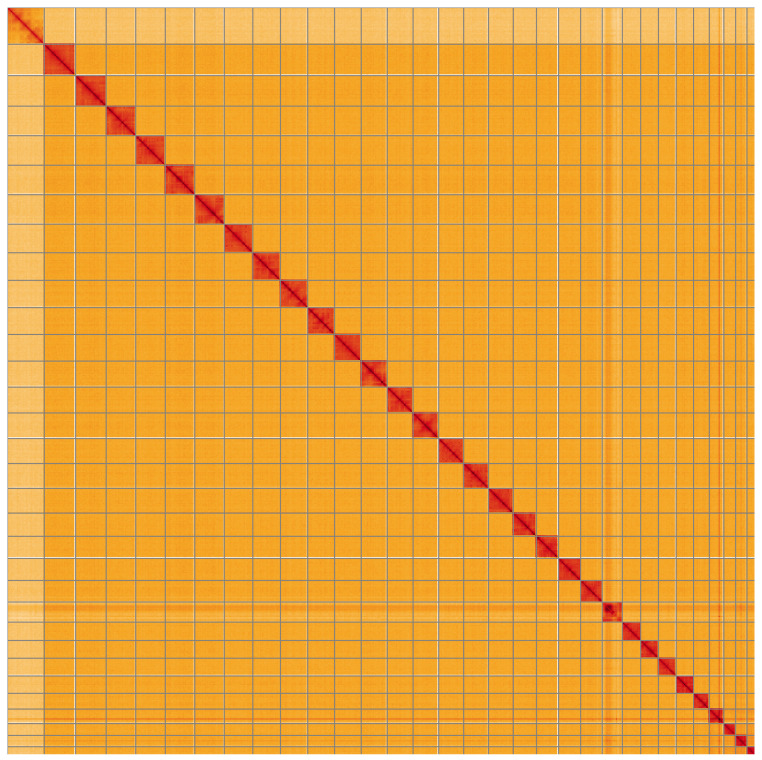
Genome assembly of *Watsonalla binaria*, ilWatBina1.1: Hi-C contact map of the ilWatBina1.1 alternate haplotype assembly, visualised using HiGlass. Chromosomes are shown in order of size from left to right and top to bottom. An interactive version of this figure may be viewed at https://genome-note-higlass.tol.sanger.ac.uk/l/?d=BZpk7k1kSBK9u8CP0jPDdg.

**Table 2.** Chromosomal pseudomolecules in the genome assembly of *Watsonalla binaria*, ilWatBina1.

INSDC accession	Chromosome	Length (Mb)	GC%
OV838918.1	1	13.92	37.0
OV838919.1	2	13.57	37.5
OV838920.1	3	13.13	37.0
OV838921.1	4	13.13	37.0
OV838922.1	5	13.11	37.5
OV838923.1	6	13.11	37.5
OV838924.1	7	12.67	37.5
OV838925.1	8	12.28	37.0
OV838926.1	9	12.13	37.0
OV838927.1	10	11.93	37.0
OV838928.1	11	11.81	37.5
OV838929.1	12	11.58	37.5
OV838930.1	13	11.46	37.0
OV838931.1	14	11.38	37.5
OV838932.1	15	11.1	37.5
OV838933.1	16	11.07	37.5
OV838934.1	17	10.99	38.0
OV838935.1	18	10.3	38.0
OV838936.1	19	9.82	38.0
OV838937.1	20	9.81	37.5
OV838938.1	21	9.56	38.0
OV838940.1	22	8.18	38.0
OV838941.1	23	7.88	38.5
OV838942.1	24	7.87	38.5
OV838943.1	25	7.71	38.0
OV838944.1	26	7.01	38.0
OV838945.1	27	6.44	39.5
OV838946.1	28	5.27	39.5
OV838947.1	29	5.0	40.0
OV838948.1	30	4.41	39.5
OV838949.1	31	0.04	47.5
OV838939.1	W	8.93	41.5
OV838917.1	Z	16.35	37.0
OV838950.1	MT	0.02	19.5

The estimated Quality Value (QV) of the final assembly is 60.5 with *k*-mer completeness of 100%, and the assembly has a BUSCO v5.3.2 completeness of 98.6% (single = 98.3%, duplicated = 0.3%), using the lepidoptera_odb10 reference set ( *n* = 5,286).

Metadata for specimens, spectral estimates, sequencing runs, contaminants and pre-curation assembly statistics can be found at https://links.tol.sanger.ac.uk/species/721165.

## Genome annotation report

The *Watsonalla binaria* genome assembly (GCA_929442735.1) was annotated using the Ensembl rapid annotation pipeline ( Table 1; https://rapid.ensembl.org/Watsonalla_binaria_GCA_929442735.1/Info/Index). The resulting annotation includes 16,183 transcribed mRNAs from 16,002 protein-coding genes.

## Methods

### Sample acquisition and nucleic acid extraction

A female *Watsonalla binaria* (ilWatBina1) was collected from Wytham Woods, Oxfordshire (biological vice-county Berkshire) (latitude 51.77, longitude –1.34) on 2020-08-01. The specimen was taken from woodland habitat by Douglas Boyes (University of Oxford),using a light trap. The specimen was identified by the collector and preserved on dry ice.

DNA was extracted at the Tree of Life laboratory, Wellcome Sanger Institute (WSI). The ilWatBina1 sample was weighed and dissected on dry ice with tissue set aside for Hi-C sequencing. Abdomen tissue was disrupted using a Nippi Powermasher fitted with a BioMasher pestle *.* High molecular weight (HMW) DNA was extracted using the Qiagen MagAttract HMW DNA extraction kit. Low molecular weight DNA was removed from a 20 ng aliquot of extracted DNA using the 0.8X AMpure XP purification kit prior to 10X Chromium sequencing; a minimum of 50 ng DNA was submitted for 10X sequencing. HMW DNA was sheared into an average fragment size of 12–20 kb in a Megaruptor 3 system with speed setting 30. Sheared DNA was purified by solid-phase reversible immobilisation using AMPure PB beads with a 1.8X ratio of beads to sample to remove the shorter fragments and concentrate the DNA sample. The concentration of the sheared and purified DNA was assessed using a Nanodrop spectrophotometer and Qubit Fluorometer and Qubit dsDNA High Sensitivity Assay kit. Fragment size distribution was evaluated by running the sample on the FemtoPulse system.

### Sequencing

Pacific Biosciences HiFi circular consensus and 10X Genomics read cloud DNA sequencing libraries were constructed according to the manufacturers’ instructions. DNA sequencing was performed by the Scientific Operations core at the WSI on Pacific Biosciences SEQUEL II (HiFi) and Illumina NovaSeq 6000 (10X) instruments. Hi-C data were also generated from head and thorax tissue of ilWatBina1 using the Arimav2 kit and sequenced on the Illumina NovaSeq 6000 instrument.

### Genome assembly, curation and evaluation

Assembly was carried out with Hifiasm ( Cheng *et al.*, 2021) and haplotypic duplication was identified and removed with purge_dups ( Guan *et al.*, 2020). One round of polishing was performed by aligning 10X Genomics read data to the assembly with Long Ranger ALIGN, calling variants with FreeBayes ( Garrison & Marth, 2012). The assembly was then scaffolded with Hi-C data ( Rao *et al.*, 2014) using YaHS ( Zhou *et al.*, 2023). The assembly was checked for contamination and corrected as described previously ( Howe *et al.*, 2021). Manual curation was performed using HiGlass ( Kerpedjiev *et al.*, 2018) and Pretext ( Harry, 2022). The mitochondrial genome was assembled using MitoHiFi ( Uliano-Silva *et al.*, 2022), which runs MitoFinder ( Allio *et al.*, 2020) or MITOS ( Bernt *et al.*, 2013) and uses these annotations to select the final mitochondrial contig and to ensure the general quality of the sequence.

A Hi-C map for the final assembly was produced using bwa-mem2 ( Vasimuddin *et al.*, 2019) in the Cooler file format ( Abdennur & Mirny, 2020). To assess the assembly metrics, the *k*-mer completeness and QV consensus quality values were calculated in Merqury ( Rhie *et al.*, 2020). This work was done using Nextflow ( Di Tommaso *et al.*, 2017) DSL2 pipelines “sanger-tol/readmapping” ( Surana *et al.*, 2023a) and “sanger-tol/genomenote” ( Surana *et al.*, 2023b). The genome was analysed within the BlobToolKit environment ( Challis *et al.*, 2020) and BUSCO scores ( Manni *et al.*, 2021; Simão *et al.*, 2015) were calculated.

Table 3 contains a list of relevant software tool versions and sources.

**Table 3.** Software tools: versions and sources.

Software tool	Version	Source
BlobToolKit	4.0.7	https://github.com/blobtoolkit/blobtoolkit
BUSCO	5.3.2	https://gitlab.com/ezlab/busco
FreeBayes	1.3.1-17-gaa2ace8	https://github.com/freebayes/freebayes
gEVAL	N/A	https://geval.org.uk/
Hifiasm	0.15.3	https://github.com/chhylp123/hifiasm
HiGlass	1.11.6	https://github.com/higlass/higlass
Long Ranger ALIGN	2.2.2	https://support.10xgenomics.com/genome-exome/ software/pipelines/latest/advanced/other-pipelines
Merqury	MerquryFK	https://github.com/thegenemyers/MERQURY.FK
MitoHiFi	2	https://github.com/marcelauliano/MitoHiFi
PretextView	0.2	https://github.com/wtsi-hpag/PretextView
purge_dups	1.2.3	https://github.com/dfguan/purge_dups
sanger-tol/genomenote	v1.0	https://github.com/sanger-tol/genomenote
sanger-tol/readmapping	1.1.0	https://github.com/sanger-tol/readmapping/tree/1.1.0
YaHS	1	https://github.com/c-zhou/yahs

### Genome annotation

The BRAKER2 pipeline ( Brůna *et al.*, 2021) was used in the default protein mode to generate annotation for the *Watsonalla binaria* assembly (GCA_929442735.1). in Ensembl Rapid Release.

### Wellcome Sanger Institute – Legal and Governance

The materials that have contributed to this genome note have been supplied by a Darwin Tree of Life Partner. The submission of materials by a Darwin Tree of Life Partner is subject to the **‘Darwin Tree of Life Project Sampling Code of Practice’**, which can be found in full on the Darwin Tree of Life website here. By agreeing with and signing up to the Sampling Code of Practice, the Darwin Tree of Life Partner agrees they will meet the legal and ethical requirements and standards set out within this document in respect of all samples acquired for, and supplied to, the Darwin Tree of Life Project.

Further, the Wellcome Sanger Institute employs a process whereby due diligence is carried out proportionate to the nature of the materials themselves, and the circumstances under which they have been/are to be collected and provided for use. The purpose of this is to address and mitigate any potential legal and/or ethical implications of receipt and use of the materials as part of the research project, and to ensure that in doing so we align with best practice wherever possible. The overarching areas of consideration are:

•   Ethical review of provenance and sourcing of the material

•   Legality of collection, transfer and use (national and international)

Each transfer of samples is further undertaken according to a Research Collaboration Agreement or Material Transfer Agreement entered into by the Darwin Tree of Life Partner, Genome Research Limited (operating as the Wellcome Sanger Institute), and in some circumstances other Darwin Tree of Life collaborators.

## Data Availability

European Nucleotide Archive: *Watsonalla binaria* (oak hook-tip). Accession number PRJEB48674; https://identifiers.org/ena.embl/PRJEB48674. ( Wellcome Sanger Institute, 2022) The genome sequence is released openly for reuse. The *Watsonalla binaria* genome sequencing initiative is part of the Darwin Tree of Life (DToL) project. All raw sequence data and the assembly have been deposited in INSDC databases. Raw data and assembly accession identifiers are reported in Table 1.

## Author information

Members of the University of Oxford and Wytham Woods Genome Acquisition Lab are listed here: https://doi.org/10.5281/zenodo.4789928.

Members of the Darwin Tree of Life Barcoding collective are listed here: https://doi.org/10.5281/zenodo.4893703.

Members of the Wellcome Sanger Institute Tree of Life programme are listed here: https://doi.org/10.5281/zenodo.4783585.

Members of Wellcome Sanger Institute Scientific Operations: DNA Pipelines collective are listed here: https://doi.org/10.5281/zenodo.4790455.

Members of the Tree of Life Core Informatics collective are listed here: https://doi.org/10.5281/zenodo.5013541.

Members of the Darwin Tree of Life Consortium are listed here: https://doi.org/10.5281/zenodo.4783558.
