# Supplementary material for: The genome sequence of the Oak Hook-tip, Watsonalla binaria (Hufnagel, 1767)
Source: Wellcome Open Res. 2023 Jul 26;8:324. [Version 1] doi: 10.12688/wellcomeopenres.19634.1 (PMC10598455; doi:10.12688/wellcomeopenres.19634.1)
Supplement: Peer review history

# Reviewer report: Reviewer response for version 1

**DOI:** 10.21956/wellcomeopenres.21750.r68217

**Published:** 2023-10-24

**Authors:** Annabel Whibley

**Affiliations:** 1 School of Biological Sciences, The University of Auckland, Auckland, Auckland, New Zealand; 2 Grapevine Improvement, Bragato Research Institute, Lincon, New Zealand

Another solid, comprehensive assembly effort and report. The assembly quality looks excellent, methods and metadata are very carefully recorded.

 K-mer completeness of 100% is impressive. This completeness is, presumably, calculated from the primary and alt assemblies combined. This could be made clearer in the methods as most other metrics are, I believe, computed just on the primary assembly.  It could be interesting to also decompose that k-mer completeness estimate to the primary and alt values too- they can give some sense of global properties of the partitions e.g. degree of heterozygosity/divergence if you're essentially recovering two complete haplotypes. Having said that, the metadata that is provided by link is well-organised, easy to navigate and would allow an interested reader to obtain exactly this kind of information.

Are sufficient details of methods and materials provided to allow replication by others?

Yes

Is the rationale for creating the dataset(s) clearly described?

Yes

Are the datasets clearly presented in a useable and accessible format?

Yes

Are the protocols appropriate and is the work technically sound?

Yes

Reviewer Expertise:

Genomics and Evolutionary Biology

I confirm that I have read this submission and believe that I have an appropriate level of expertise to confirm that it is of an acceptable scientific standard.

# Reviewer report: Reviewer response for version 1

**DOI:** 10.21956/wellcomeopenres.21750.r68216

**Published:** 2023-10-10

**Authors:** Benoit Nabholz

**Affiliations:** 1 Institut des Sciences de l'Evolution de Montpellier, Universite de Montpellier, Montpellier, Occitanie, France

The authors present the chromosome scale assembly of the Oak Hook-tip, *Watsonalla binaria*. Eleven genomes are currently available for the family Drepanidae. All have been produced by the Darwin Tree of Life project (DToL).

 The genome is of high quality and the pipeline is the same as for the other genomes produced by the DToL. All links and datasets presented are publicly available.

 Again, the photographs of the samples could have been much better. Also, there is no indication if the specimen (at least what is left of it) is kept in a collection (i.e. a voucher specimen).

 The text of the introduction reads well, but very little information is provided. Perhaps the DtoL should consider publishing several genome in one note. For example, regrouping several species from the same family or simply several moths. All these notes are starting to become very redundant and I'm not sure if anyone is actually reading them.

Are sufficient details of methods and materials provided to allow replication by others?

Yes

Is the rationale for creating the dataset(s) clearly described?

Yes

Are the datasets clearly presented in a useable and accessible format?

Yes

Are the protocols appropriate and is the work technically sound?

Yes

Reviewer Expertise:

Molecular evolution

I confirm that I have read this submission and believe that I have an appropriate level of expertise to confirm that it is of an acceptable scientific standard.
